# The dicot homolog of maize PPR103 carries a C-terminal DYW domain and is required for C-to-U editing of chloroplast RNA transcripts

**DOI:** 10.21203/rs.3.rs-2574001/v1

**Published:** 2023-02-23

**Authors:** Tyra N. McCray, Mohammad F. Azim, Tessa M. Burch-Smith

**Affiliations:** a School of Genome Science and Technology, University of Tennessee, Knoxville, TN 37996; b Department of Biochemistry and Cellular & Molecular Biology, University of Tennessee, Knoxville, TN 37996; c Donald Danforth Plant Science Center, St. Louis, MO 63132

**Keywords:** chloroplast gene expression, C-to-U editing, DYW domain, *Nicotiana benthamiana* pentatricopeptide repeat, PPR103, RNA editing

## Abstract

In plants, cytidine-to-uridine (C-to-U) editing is a crucial step in processing mitochondria and chloroplast-encoded transcripts. This editing requires nuclear-encoded proteins including members of the pentatricopeptide (PPR) family, especially PLS-type proteins carrying the DYW domain. *IPI1/emb175/PPR103* is a nuclear gene encoding a PLS-type PPR protein essential for survival in *Arabidopsis thaliana* and maize. Arabidopsis IPI1 was identified as likely interacting with ISE2, a chloroplast-localized RNA helicase associated with C-to-U RNA editing in Arabidopsis and maize. Notably, while the Arabidopsis and *Nicotiana* IPI1 homologs possess complete DYW motifs at their C-termini, the maize homolog, ZmPPR103, lacks this triplet of residues which are essential for editing. We examined the function of ISE2 and IPI1 in chloroplast RNA processing in *N. benthamiana*. A combination of deep sequencing and Sanger sequencing revealed C-to-U editing at 41 sites in 18 transcripts, with 34 sites conserved in the closely related *N. tabacum*. Virus induced gene silencing of *NbISE2* or *NbIPI1* led to defective C-to-U revealed that they have overlapping roles at editing a site in the *rpoB* transcript but have distinct roles in editing other transcripts. This finding contrasts with maize *ppr103* mutants that showed no defects in editing. The results indicate that NbISE2 and NbIPI1 are important for C-to-U editing in *N. benthamiana* chloroplasts, and they may function in a complex to edit specific sites while having antagonistic effects on editing others. That NbIPI1, carrying a DYW domain, is involved in organelle C-to-U RNA editing supports previous work showing that this domain catalyzes RNA editing.

## INTRODUCTION

In land plant organelles, post-transcriptional processing of RNA transcripts is a crucial regulatory point for gene expression. One step of post-transcriptional RNA processing is the site-specific deamination of cytidine to uridine, called C-to-U editing. In most land plants this C-to-U RNA editing occurs within a subset of chloroplast and mitochondrial transcripts and editing of these sites often produces changes in splice sites, amino acid substitutions and the start and stop codons within transcripts. These modifications yield transcripts that can subsequently be translated to produce proteins essential for photosynthesis and for mitochondrial function (Small et al. 2020). C-to-U editing involves numerous nucleus-encoded proteins including several families of RNA binding proteins.

The largest family of plant proteins with roles in post-transcriptional processing and C-to-U editing of organelle transcripts is the pentatricopeptide repeat (PPR) protein family. PPR proteins are site-specific RNA-binding proteins that have critical and diverse functions (Saha et al. 2007; Schmitz-Linneweber and Small 2008; Barkan and Small 2014; Small et al. 2020;). Loss of a single PPR protein can result in embryonic arrest or severe developmental defects, demonstrating their fundamental importance to plant survival and development (Barkan and Small 2014). Plant PPR proteins are typically targeted to either the mitochondria or chloroplast, where they act by binding to one or several single-stranded RNA molecules via 2–30 N-terminal tandem helical repeat motifs (Shikanai 2006). Within plant mitochondria and chloroplasts, most characterized PPR proteins mediate specific events in post-transcriptional processing and maturation of RNA by influencing RNA splicing, RNA cleavage, RNA stability, translation and the site-specific sequence alteration of RNA transcripts through a process called RNA editing [reviewed in (Barkan and Small 2014)]. Other cellular processes that are affected by PPR proteins include nuclear gene expression ( Ding et al. 2006; Koussevitzky et al. 2007; Liu et al. 2010) and plastid biosynthesis (Beick et al. 2008).

PPR proteins are divided into the P subfamily and the plant-specific PLS subfamily, and both classes of PPR proteins have diverse roles in RNA metabolism. In *Arabidopsis thaliana*, the P class comprises around half of the 450 known PPR proteins. While the P family proteins contain only PPR repeats, the PLS subfamily is further subdivided according to the domains present in their extended C-terminal regions: (i) E proteins contain an E domain as the C-terminal region (ii) E+ proteins carry an E domain and an E+ C-terminal region, and (iii) DYW proteins possess an E, E+ and an additional domain named the DYW domain due to the presence of aspartic acid, tyrosine, and tryptophan triplet of amino acids (DYW), or a variation thereof, at their extreme C-termini (Shikanai 2006). The DYW domain appears to be plant specific and has sequence similarity to cytidine deaminases (Salone et al. 2007; Iyer et al. 2011), enzymes involved in the recognition of target cytidines in the C-to-U editing reaction (Okuda et al. 2014). The PPR DYW domain has been correlated with the occurrence of RNA editing, while the E domains are thought to recruit editing enzymes (Schmitz-Linneweber and Small 2008). PPR proteins within the PLS subfamily with functional roles in RNA editing all belong to the E or DYW PPR subfamilies (Shikanai 2006).

The RNA editing factor interacting proteins (RIP)/Multiple organellar RNA editing factor (MORF) proteins are a small family of proteins required for C-to-U editing in both chloroplasts and mitochondria (Takenaka et al. 2012; Bentolila et al. 2013). RIP/MORF2 and 9 are required for editing of chloroplast transcripts while the other proteins are involved in mitochondrial editing (Takenaka et al. 2012) and RIP1/MORF8 is dual targeted to chloroplasts and mitochondria (Bentolila et al. 2012). RIP/MORF proteins carry a novel conserved domain of about 100 amino acids, the MORF box, that is required for multimerization and interaction with PPR proteins (Takenaka et al. 2012; Bayer-Csaszar et al. 2017; Haag et al. 2017; Yan et al. 2017; Yang et al. 2018). MORF proteins interact with PPR proteins (Bentolila et al. 2012; Takenaka et al. 2012; Glass et al. 2015; Yan et al. 2017;) to induce structural changes in that increase RNA-binding and hence editing efficiency (Yan et al. 2017). While PPR proteins are usually involved in editing of a few sites, loss of some RIP/MORF proteins can cause defects in editing of all sites in chloroplasts (RIP/MORF2 and 9) (Takenaka et al. 2012) or hundreds of sites in mitochondria (Bentolila et al. 2012). Biochemical evidence has been interpreted as indicating that an RNA helicase is a likely component of the organellar RNA editing machinery (Takenaka and Brennicke 2003; Hegeman et al. 2005). Editing activity in chloroplast extracts could be stimulated by ATP, CTP or dCTP (Hegeman et al. 2005) and similarly, mitochondrial extracts from pea that were used for in vitro editing assays could use any NTP or dNTP (Takenaka and Brennicke 2003). Consistent with the proposed involvement of an RNA helicase in organelle RNA editing, the chloroplast RNA helicase ISE2 was shown to be required for editing of several different Arabidopsis chloroplast transcripts (Bobik et al. 2017). The involvement of ISE2 in chloroplast C-to-U editing is supported by the identification of the maize ISE2 orthologue in RIP9 complexes purified from maize extracts (Sandoval et al. 2019), indicating that ISE2 is involved in editing in multiple plants. In studies to determine ISE2’s involvement in chloroplast RNA processing, we identified protein partners of ISE2 including a DYW protein encoded by *At5g03800/EMB175* as a potential partner of ISE2 and we later named it ISE2 PROTEIN INTERACTOR1 (IPI1) (Bobik et al. 2019; Ganusova et al. 2020). The Arabidopsis *embryo defective 175* (*emb175*) mutant embryos arrest at the globular-heart transition (Cushing et al. 2005). The maize ortholog of IPI1/EMB175, PPR103, functions in rRNA processing and stabilization, and loss of PPR103 resulted in seedling lethality (Hammani et al. 2016).

Over the last two decades *Nicotiana benthamiana* has gained prominence as an excellent model for studying varying aspects of plant biology ( Bally et al. 2018; Goodin et al. 2008). It has been critical for examining plant-virus interactions, plant immunity and other plant-microbe interactions. *N. benthamiana* belongs to the family Solanaceae and its evolutionary closeness to important Solanaceous crops like tomato and potato has also contributed to its popularity as a model plant. Another feature of *N. benthamiana* that has sped its adoption for experimental biology is the efficacy of virus-induced gene silencing (VIGS) as a tool for reverse genetics in this plant. A major advantage of VIGS is that it can be used to study genes that are essential for survival in other plant models, since the silenced tissues are supported by non-silenced tissues on the same plant, enabling the survival of the silenced tissues (Burch-Smith et al. 2004). Tobacco (*N. tabacum*) is a close relative of *N. benthamiana* that has been used to characterize C-to-U editing in the organelles of land plants (Hirose et al. 1999; T. Sasaki et al. 2003). We therefore set out to investigate the occurrence of C-to-U RNA editing in the model plant *N. benthamiana* and to identify host factors involved.

Sanger sequencing of *N. benthamiana* chloroplast transcripts known to contain C-to-U editing sites in tobacco combined with deep sequencing of the entire *N. benthamiana* chloroplast transcriptome revealed conservation of editing between the two species and identified three editing sites in *N. benthamiana* transcripts that had not been reported in tobacco. Consistent with editing in tobacco, editing in *N. benthamiana* was also found to be a function of leaf tissue developmental age. Tobacco rattle virus (TRV)-mediated VIGS was used to investigate the function of the *N. benthamiana* homologs of *ISE2* and *IPI1/EMB175/PPR103* (hereafter *NbIPI1*). VIGS avoided the embryonic and seedling lethality associated with loss of ISE2 in Arabidopsis and PPR103 in maize, respectively (Kobayashi et al. 2007; Hammani et al. 2016). Here we show that *NbISE2* and *NbIPI1* function in C-to-U editing, and VIGS of each gene had unique but partially overlapping effects on editing chloroplast transcripts. Of note is that while ZmIPI1/PPR103 lacks the C-terminal DYW triplet of amino acids typical to DYW proteins and was reported to not have a role in chloroplast C-to-U RNA editing, silencing *NbIPI1* which carries the C-terminal DYW triplet and has a complete DYW motif resulted in modified editing of several sites.

## MATERIALS AND METHODS

### Plant Materials and Growth Conditions

*Nicotiana benthamiana* seedlings were grown on a light cart at 25°C under fluorescent white light in a 16:8-hour light/dark cycle. Around 1-week old seedlings were transplanted to individual pots and typically silenced using VIGS at around 2–3 weeks of age.

### RNA Editing by Sanger sequencing

For editing in the non-silenced control, *ISE2-*, *IPI1-* or *PDS*-silenced leaves, RNA was isolated from leaf number 11 of approximately six-week-old plants using Trizol (Thermo Fisher Scientific, Waltham, MA). The RNA was treated with DNase (30 minutes with 0.5 μL rDNase, 15 minutes of a 2^nd^ 0.5μl rDNase at 37°C) at least once. RT-PCR was conducted according to manufactures instructions in the M-MLV RT (Promega, Madison, WI,) manual using random primer hexamers. A typical reaction consisted of PCR: 1 μg RNA, 1.2 μL random hexamer, 0.8 μL reverse transcriptase. The same reaction without the reverse transcriptase was performed in parallel with the experimental cDNA synthesis reaction to ensure the absence of genomic DNA contamination. Second-strand PCR synthesis was performed according to standard Taq polymerase protocol using primers in Table S3. PCR was conducted with an annealing temperature of 53 for almost all primer pairs. PCR products were gel purified using the Gel Extraction Wizard kit (Promega, Madison, WI). Purified amplicons were sequenced by the UTK Genomics Core Facility.

For examination of editing in 6-week-old WT *N. benthamiana* plants, plants were grown under standard growth conditions leaves 4,6 and 8 were snap frozen in liquid nitrogen. RNA was isolated using Trizol (Thermo Fisher Scientific, Waltham, MA) and RT-PCR was conducted using Moloney Murine Leukemia Virus Reverse Transcriptase (M-MLV RT) (Promega, Madison, WI) according to manufacturer’s instructions. PCR was conducted on cDNA to amplify specific transcripts containing editing sites of interest. PCR products were gel purified using Wizard gel purification kit (Promega, Madison, WI), diluted to about 5ng per 100 bp and sent to sequencing. Three individual biological replicates were analyzed. The same cDNA used for editing experiments was also used to assay *NbIPI1* expression using qPCR.

### VIGS constructs and protocol

Tobacco rattle virus (TRV)-based VIGS constructs used for the non-silencing control and silencing *ISE2* and *IPI1* were described previously (Burch-Smith and Zambryski 2010; Ganusova et al. 2020). Constructs were transformed into *Agrobacterium tumefaciens* GV3101 (pMP90RK) strain. VIGS of *IPI1*, *ISE2*, *GUS* intron (negative control) and *PDS* (positive control) were performed according to (Burch-Smith and Zambryski 2010; Ganusova et al. 2020). Approximately 3-week-old *N. benthamiana* plants were infiltrated with the respective constructs and then grown under standard growth conditions until downstream assays were performed.

### Transient expression and Confocal Microscopy

Leaves of 5–6-week-old plants were agroinfiltrated with constructs for expression of AtIPI1-YFP, cTP-AtIPI1-YFP or cTP-NbIPI-YFP. Forty-eight hours later infiltrated leaf sections were vacuum infiltrated with water and mounted on slides for imaging. Confocal fluorescence microscopy was performed using a Leica SP2 or SP8X confocal laser scanning microscope (Leica Microsystems, Heidelberg GmbH). A 40x or 63x HCX PL APO objective was used for image acquisition. The samples were excited with an excitation line of 458/514 nm for YFP. The numerical aperture of the objective was 1.32.

### Transmission Electron Microscopy (TEM)

Samples were prepared for TEM as described previously (Burch-Smith and Zambryski 2010; Burch-Smith et al. 2011). Briefly, samples from young emerging leaves from control or *IPI1-*silenced plants were fixed by high-pressure freezing (HPF) and quick freeze substituted (QFS) in 1% osmium tetroxide plus 0.1% uranyl acetate in acetone. Subsequently, samples were embedded in epoxy resin Embed 12, (Ted Pella, Inc., Redding, CA), sliced into ultrathin 65–70-nm sections, and visualized on a Libra 200M TEM/STEM (Carl Zeiss Microscopy, White Plains, NY) at 200 kilovolts.

### Prediction of NbIP1 binding sites

Potential binding sites for NbPPR103/IPI1 were predicted with the FIMO program in the MEME suite (Hirose et al. 1999; Sasaki et al. 2003; Grant et al. 2011). The nucleotide-binding probabilities for NbPPR103/IPI1 were generated based on the amino acids found at the 6 and 1’ position (first amino acid of the subsequent C terminal PPR motif) of each PPR motif to assign a nucleotide preference according to the weighting scheme in (Takenaka et al. 2013). These nucleotide preferences scores were used to predict NbPPR103/IPI1 RNA binding sites within the *Nicotiana benthamiana* chloroplast genome using the FIMO program. The ten top-predicted binding sites were ranked by *P-*values calculated by FIMO (Grant et al. 2011).

### Chloroplast Isolation and RNAseq library preparation

Chloroplasts were extracted according to “Extraction of Chloroplast Proteins from Transiently Transformed *Nicotiana benthamiana* Leaves” bio protocol (Klinkenberg 2014; Klinkenberg et al. 2014). Briefly, fresh leaf tissue was ground, filtered and centrifuged through a Percoll gradient and visualized on an inverted microscope. Chloroplasts were then shock-frozen and total RNA was isolated from purified chloroplasts using Trizol (Thermo Fisher Scientific, Waltham, MA) or RNeasy Plant Mini kit (Qiagen, Germantown, MD) as per manufacturers’ instructions. For each plant, approximately 100 mg of tissue was ground from each leaf to isolate chloroplast RNA. Leaves from individual plants were pooled. Removal of chloroplast DNA was done by treating the samples with Ambion rDNase1 (Thermo Fisher Scientific, Waltham, MA). Because rRNA typically constitutes over 75% of total RNA and its depletion can results in very low yields of RNA for cDNA preparation, rRNA depletion was not performed. The RNA integrity of the isolated RNA was examined on a Bioanalyzer machine and quantitated on a NanoDrop 1000 spectrophotometer (Thermo Scientific, Waltham, MA) prior to library preparation. For cDNA synthesis, about one microgram of non rRNA-depleted RNA was used to make double strand cDNA (ds-cDNA) and dsDNA was produced using the Invitrogen SuperScript II Double Stranded cDNA Synthesis kit (Thermo Fisher Scientific, Waltham, MA) with random hexamers primers for first-strand synthesis. The cleaned ds-cDNA was then used to construct a library using the Illumina Next Tera Library prep kit with no adaptations (Illumina, Inc, San Diego, CA). After examination of the library quality using the Bioanalyzer (Agilent, Santa Clara, CA), multiplexed libraries were sequenced using the Illumina MiSeq sequencing platform per standard MiSeq run parameters (Illumina protocol manuals).

### Mapping and Data Statistical Analysis

Sequence reads were examined for sequence quality, trimmed using the base space graphical user app (Base Space, Illumina, Inc) and mapped to the *N. benthamiana* genome (NC) using DNA Array Star Next Gen Seq software (version 12) permitting multiple mismatches to detect multiple SNPs. The mapping parameters utilized kmer size of 21 and low SNP filter stringency (to avoid missing highly edited transcripts). Paired-end mapped contigs were visualized in Seq Man Pro software or the Integrative Genomics Viewer (IGV) software. The SeqMan NGen-mapped contigs (Supplementary Table S2) were used for subsequent analysis. The uniquely mapped reads were used to detect coverage information for each sample. The coverage summary additionally reveals the depth of sequenced reads that were mapped at each locus in the *Nicotiana* genome. Overall, a similar number of reads were mapped to the *N. benthamiana* genome in all samples.

### Northern Blotting

One microgram of total RNA was run on a denaturing formaldehyde gel, transferred to positively charged Roche nylon membranes (MilliporeSigma, Burlington, MA), and hybridized with DIG-labeled 23S rRNA and 5S rRNA probes (Table S3) according to manufacturer’s instructions (PCR DIG Probe Synthesis Kit, Roche). Bands corresponding to ribosomal RNA species were detected using the Roche DIG High Prime DNA Labeling and Detection Starter Kit II (MilliporeSigma, Burlington, MA). The same RNA that was used to measure C-to-U editing efficiencies was used for the Northern Blot analysis.

## RESULTS

### Chloroplast C-to-U RNA editing in N. benthamiana

**Fig. 1:**
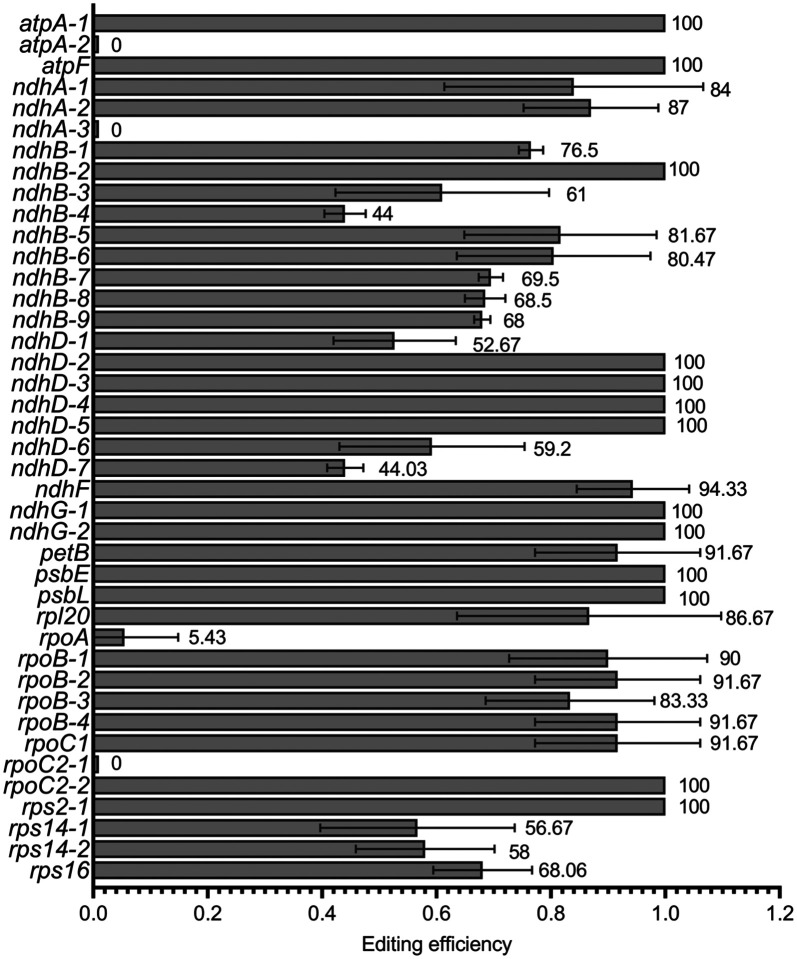
C-to-U RNA editing in *N. benthamiana* chloroplasts. Chloroplast transcripts reported to undergo C-to-U editing in other Nicotiana species were sequenced by the Sanger method and editing efficiency is represented as a fraction calculated as T/(C+T) (horizontal axis), and labels over individual bars present the calculated per cent editing for a given site. Sites are named according to (Hirose et al. 1999). The mean of two or three independent biological replicates are presented and error bars represent one standard deviation

C-to-U RNA editing of chloroplast transcripts has been catalogued for several *Nicotiana* species including *N. tabacum* (Hirose et al. 1999; Sasaki et al. 2003; Sasaki et al. 2006), *N. tomentosiformis* and *N. sylvestris* (T. Sasaki et al. 2003); *N. glutinosa*, *N. rustica*, *N. knightiana*, *N. paniculata, N. obtusifolia,* and *N. glauca* (Hirose et al. 1999; Mehmood et al. 2020). We hypothesized that patterns of editing would be similar between these species and *N. benthamiana* given their evolutionary relationships. Based on these previous results we took a candidate approach and examined the degree of editing of probable conserved sites, by Sanger bulk sequencing of cDNA made from RNA extracted from pooled leaves from 6-week-old plants confirmed editing of 41 sites in 18 transcripts (Supplementary Figure S1). Note that sites are named after the convention of Hirose et al. (1999). Of the 41 sites examined, editing was observed at 38 sites with only *atpA-2*, *ndhA-3*, and *rpoC2–1* showing no editing (Fig. 1). For the edited sites, editing efficiencies ranged from 1 (100%; *atpA-1*, *atpF*, *ndhB-2*, *ndhD-2–6*, *ndhG-1* and *−2*, *psbE*, *psbL*, *rpoC2–2* and *rps2–1*) to as low as 0.05 (5.43% for *rpoA*). Consistent with previous reports, the *ndhB* transcript is extensively edited with 9 sites edited and all were edited to extents greater than 0.4 (Fig. 1). In summary, the analysis reveals that editing is largely conserved among *N. benthamiana* and the other *Nicotiana* species examined to date (Supplementary Table S1), although the degree of editing at different sites was more variable in *N. benthamiana* than in other species although this may be due technical disparities between experiments.

### C-to-U RNA editing of specific transcripts in N. benthamiana chloroplasts changes during development

**Fig. 2:**
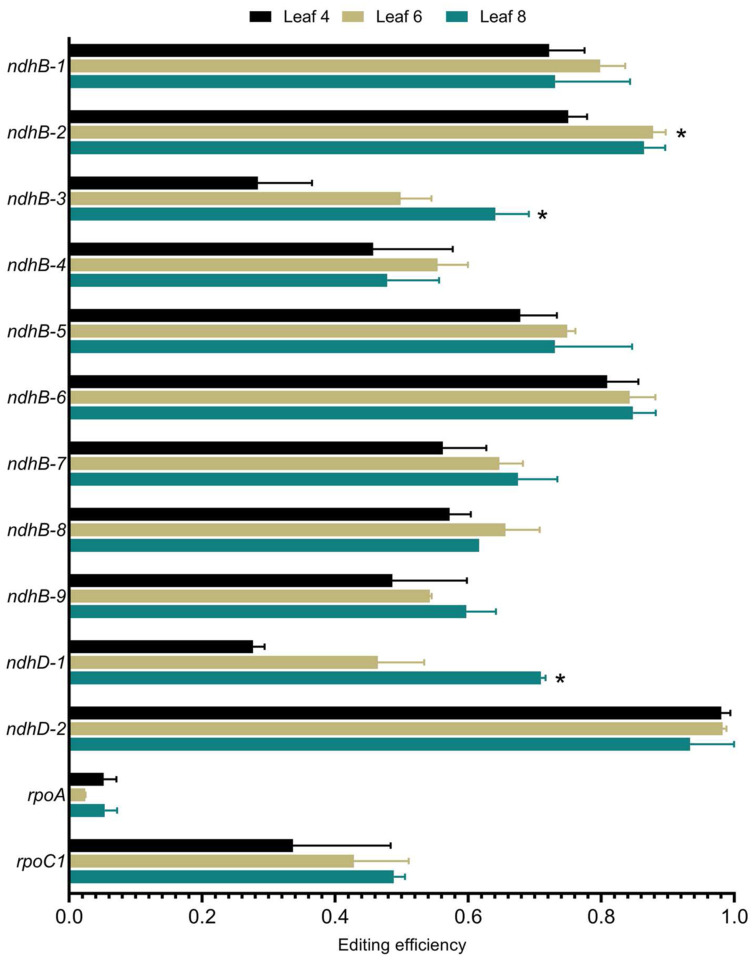
Developmental dependence of chloroplast C-to-U RNA editing in *N. benthamiana*. Editing efficiency of select sites was measured in leaves of different ages and revealed a general trend of decreased editing in older leaves (leaf 4) compared to younger leaves (leaf 8). Error bars represent standard deviation from two independent biological samples. Statistical significance was measured by Student’s *t*-test and asterisks represent p-values <0.05

Editing of chloroplast transcripts varies in response to development and environmental signals (Hirose et al. 1996; Karcher and Bock 2002; Tseng et al. 2013; Tian et al. 2019). To examine the effects of these factors on C-to-U editing of RNA transcripts in *N. benthamiana* editing of thirteen sites from four chloroplast transcripts from leaves at different developmental stages was examined by Sanger sequencing. Reduced editing efficiency of several sites in chloroplasts from older leaves (leaf 4) compared to those from younger leaves (leaf 8; Fig. 2). This is most pronounced for sites *ndhB-3* and *ndhD-1*, while other sites show almost no dependence with leaf age, exemplified by *ndhB-6* and *ndhD-2*. Thus, like in tobacco, chloroplast C-to-U RNA editing depends on the developmental state of the tissue (Hirose et al. 1996; 1999; Karcher and Bock 2002).

### The RNA helicase ISE2 has a role in C-to-U RNA editing in N. benthamiana chloroplasts

**Fig. 3:**
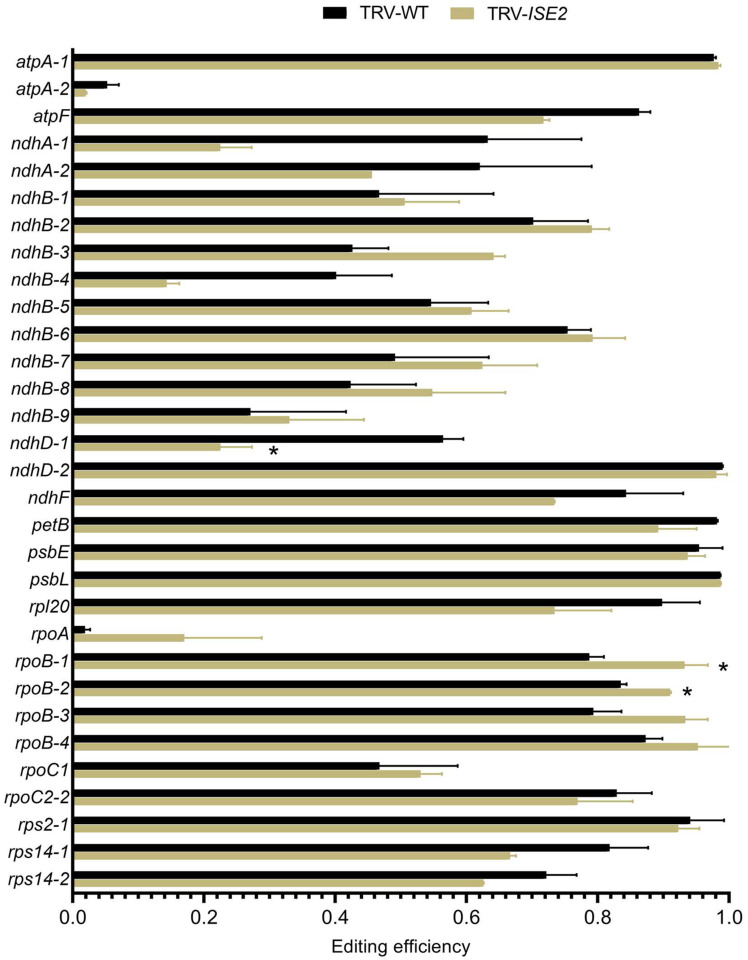
Knockdown of ISE2 by VIGS changes chloroplast RNA editing in *N. benthamiana*. Editing efficiency of chloroplast transcripts in *ISE2*-silenced plants compared to TRV-infected, non-silenced controls was measured by Sanger sequencing. Error bars represent standard deviation from two or three independent biological replicates. Statistical significance was measured by Student’s *t*-test and asterisks represent p-values <0.05

Previous work in *Arabidopsis thaliana* revealed reduced editing for 12 of 34 edited sites in chloroplast transcripts from *ISE2*-silenced leaves with ISE2 being specifically required for editing three sites (Bobik et al. 2017). ISE2 was identified as a constituent of a maize protein complex that mediates C-to-U editing (Bobik et al. 2019; Sandoval et al. 2019), implicating ISE2 in chloroplast C-to-U editing in diverse plants. We therefore tested the role of *NbISE2* in chloroplast editing. Tobacco rattle virus (TRV)-mediated VIGS was used to knockdown *NbISE2* expression in *N. benthamiana* leaves and C-to-U editing at 18 sites in 12 chloroplast transcripts was measured by Sanger sequencing. Plants of the same age infected with TRV were used as the non-silenced control. At 10 of the 31 sites where editing was measured, *atpA-2*, *atpF*, *ndhA-1* and *−2*, *ndhB-4*, *ndhD-1*, *ndhF*, *rpl20*, *rps14–1* and *−2*, silencing *NbISE2* led to decreased editing efficiency although this was only statistically significant for *ndhD-1* (Fig. 3). For six sites, *ndhB-3*, *rpoA* and *rpoB-1–4*, editing efficiency was increased in *NbISE2*-silenced plants compared to the non-silenced controls, and this was statistically significant for *rpoB-1* and *−2* (Fig. 3). These results suggest that ISE2 may affect editing efficiency differently in Arabidopsis and *N. benthamiana* chloroplasts. It appears that ISE2 promotes C-to-U editing in Arabidopsis while it can both promote and inhibit editing in *N. benthamiana*. The reason for the different effects of ISE2 on the Arabidopsis and *N. benthamiana* editing reactions is unclear but may be due to species-specific differences in the presence and/or amounts of other regulatory editing machinery components.

### The chloroplast PPR protein, NbIPI1, is needed for chloroplast development in N. benthamiana

**Fig. 4:**
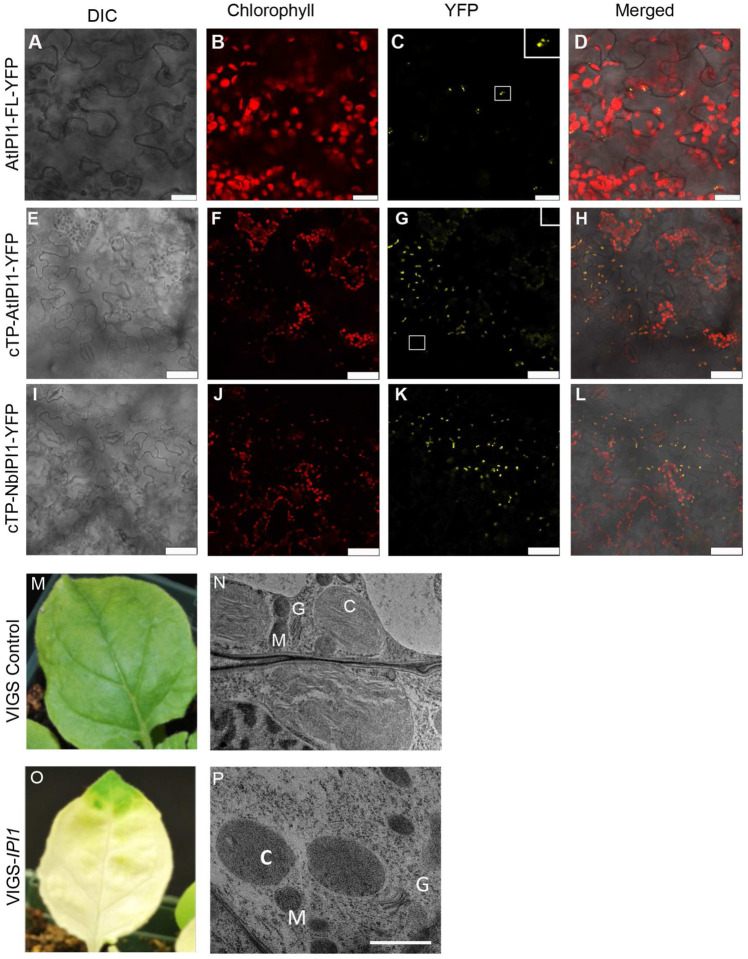
Subcellular localization of IPI1 and effects of NbIPI1 knockdown on leaves and chloroplasts. (**A-D**) AtIPI1-YFP was transiently expressed in *N. benthamiana* leaves and YFP signal overlapped with chloroplast autofluorescence. Scale bars represent 20 μm. Inset shows enlarged image of boxed region. (**E-F**) The predicted cTP of AtIPI1 was fused to YFP and transiently expressed in *N. benthamiana* leaves. Inset shows enlarged image of boxed region. Scale bars represent 50 μm. (**G-H**) The predicted cTP of NbIPI1 was fused to YFP and transiently expressed in *N. benthamiana* leaves. Scale bars represent 50 μm. (**M-N**) TRV-infected, non-silenced control leaves, and TEM image showing chloroplasts in young sink leaves with forming thylakoids and grana. C, M, and G indicate chloroplast, mitochondria, and Golgi, respectively. (**O-P**) *IPI1*-silenced leaves presented a severe chlorotic phenotype and TEM analysis reveals defective chloroplasts. Scale bar represents 1μm

We had identified the PPR protein encoded by *At5g03800/emb175/IPI1* as interacting with AtISE2 in a yeast two-hybrid screen (Bobik et al. 2019). To confirm the subcellular localization of IPI1, the full-length Arabidopsis *IPI1* coding sequence was cloned upstream of the yellow fluorescent protein (YFP). The resulting fusion protein (AtIPI1-YFP) was transiently expressed in *N. benthamiana* leaves and visualized by confocal laser scanning fluorescence microscopy. Fluorescence from the IPI-YFP fusion colocalized with chloroplast autofluorescence, indicating that AtIPI1-YFP localized to chloroplasts (Fig. 4a–d). AtIPI1-YFP also localized to punctae within the chloroplast, suggesting that it may localize to the chloroplast stroma. A similar pattern of fluorescence was observed when the predicted chloroplast targeting peptide (cTP) plus 20 amino acids downstream of the cTP were cloned as a translation fusion to YFP (cTP-AtIPI1-YFP; Fig. 4 e–h). To determine NbIPI1’s subcellular localization, a similar construct carrying the predicted cTP of NbIPI was introduced into *N. benthamiana* leaves for transient expression. Fluorescence from cTP-NbIPI1-YFP co-localized with chloroplast autofluorescence, indicating that the fusion was imported into chloroplasts and that NbIPI1 localizes to the chloroplast stroma (Fig. 4i–l).

Arabidopsis *emb75* mutants fail to develop past the very early stage of embryonic development, arresting at the globular embryo stage (Cushing et al. 2005). VIGS of *NbIPI1* in *N. benthamiana* caused severe leaf chlorosis and reduced chlorophyll content [(Fig. 4m and o); and (Ganusova et al. 2020)]. Transmission electron microscopy on young sink leaves from silenced plants revealed profound effects of *IPI1* knockdown on chloroplast development. While thylakoids and nascent grana were observed in TRV-infected non-silenced controls (Fig. 4n), these structures were largely absent from chloroplasts in *NbIPI1*-silenced leaves (Fig. 4p). These observations are consistent with IPI1’s localization to chloroplasts (Fig. 4a–l) and severe chlorosis of *NbIPI1*-silenced leaves (Fig. 4o) and suggest that NbIPI1 has critical roles in chloroplast development.

### IPI1 orthologues have divergent sequences hinting at differing functions.

**Fig. 5:**
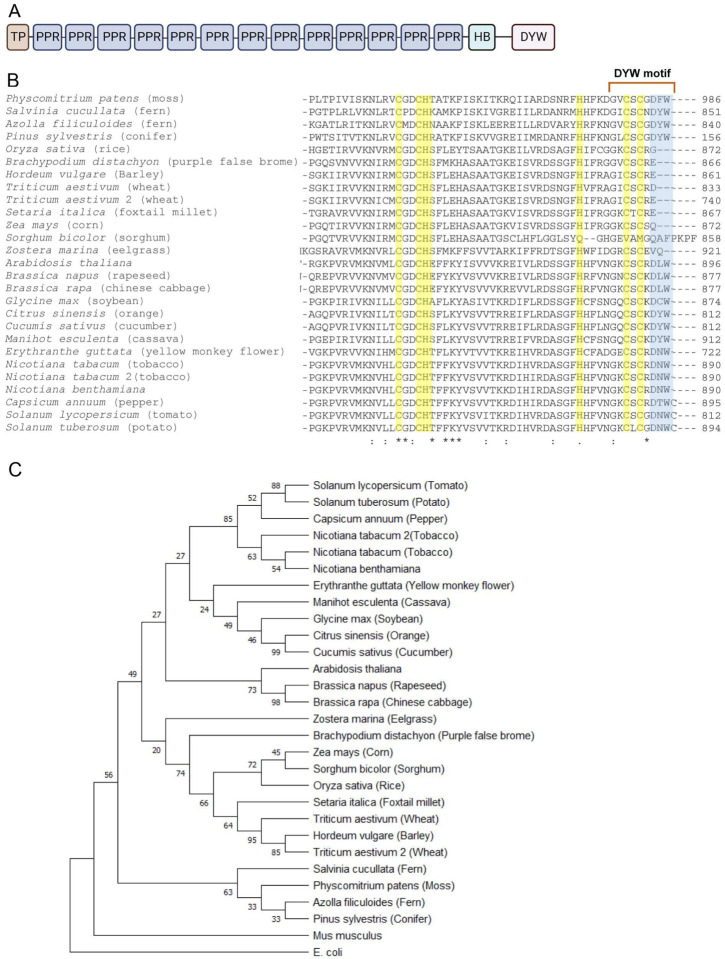
NbIPI1 domain structure and evolutionary relationships. (**A**) The predicted domains of NbIPI1 including chloroplast targeting peptide (cTP), PPR domains, heme-binding domain (HB) and DYW domain (DYW). (**B**) Amino acid sequence alignment of the C-terminal region of select NbPPR103/IPI1 orthologs. DYW triplet is shown in blue shading. Asterisks, colons, and periods indicate identical amino acid residue, conserved substitution, and semi-conserved amino acids, respectively. Residues for shaded in yellow are Zn binding. (**C**) The phylogenetic tree was generated using the amino acid sequences of NbPPR103/IPI1 orthologs by the Neighbor-Joining method. The bootstrap values calculated as per million for 1,000 replications are shown at nodes. The evolutionary distances were computed using the p-distance method and are in the units of the number of amino acid differences per site

NbIPI1 is predicted to comprise a plastid-localization sequence at its N-terminus, 13 PLS PPR motifs and C-terminal E and DYW domains (Fig.5a and Supplemental Figure S2). The DYW domain is named after the C-terminal aspartate, tyrosine and tryptophan tripeptide that is often found in PPR proteins associated with C-to-U RNA editing in organelles (Gutmann et al. 2020). Recent findings revealed that the DYW domain catalyzes the C-to-U editing through a unique mechanism (Takenaka et al. 2021). Given the importance of the Zinc binding and DYW domains we examined the C-termini (containing part of the DYW domain) of IPI1 orthologues from diverse plants. Sequence alignment revealed changes in the C-termini of this PPR protein in different evolutionary lineages (Fig. 5b). In all orthologues examined, the residues involved in Zinc binding (highlighted in yellow) are highly conserved. In early land plants including mosses and ferns, the DYW motif costing of 9 C-terminal residues, as previously defined ( Takenaka et al. 2021), is also present (Fig. 5B). The DYW motif is also present in dicots represented in the alignment, where the tyrosine residue is most often substituted by a leucine as in Arabidopsis (DLW) or asparagine residue as in *N. benthamiana* (DNW), (Fig. 5B and Supplemental Figure S2). Interestingly, the C-terminal DYW triplet is absent from the DYW motifs of the examined monocots, as has been reported for maize [Fig. 5b, (Hammani et al. 2016)]. This finding is supported by phylogenetic analysis, where the monocot proteins form a distinct clade (Fig. 5c). The absence of the DYW motif from the maize IPI orthologue, PPR103, likely explains previous observations that suggest it is not involved in C-to-U RNA editing of maize chloroplast transcripts (Hammani et al. 2016).

### Identification of predicted NbIPI1 targets

**Fig. 6:**
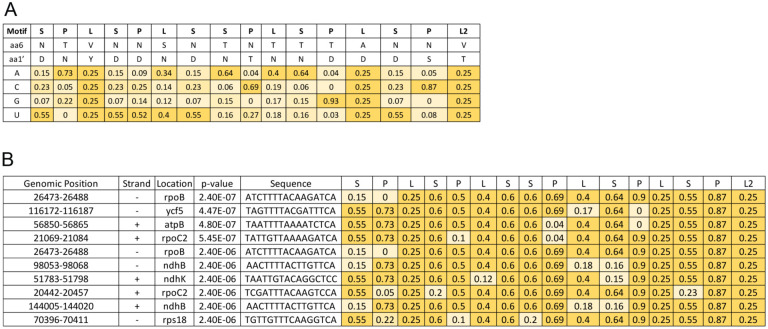
PPR code-based prediction of NbIPI1 binding sites. (**A**) Nucleotide-binding probabilities for NbIPI1 motifs (P, L, and S) based on the amino acids found at the 6 and 1′ position (first amino acid of the subsequent C terminal PPR motif) of each PPR motif (see Supplementary Figure S1). (**B**) Prediction of NbIPI1 binding sites within *N. benthamiana* chloroplast genome. The ten top-ranking matches are shown. The genomic location and nucleotide sequence of each site are indicated, along with the binding score for each repeat. The *P-*values were calculated with the FIMO program

Given that the DYW domain is essential for C-to-U editing, we hypothesized that dicot IPI1 proteins may retain catalytic activity in editing, in contrast to the monocot orthologs. We therefore examined whether C-to-U RNA editing was affected in plants where expression of *IPI1* was perturbed. We began this analysis by taking advantage of publicly available software tools for predicting targets of PPR proteins (Barkan et al. 2012; Takenaka et al. 2013; Kobayashi et al. 2019). Computationally predicted sites for Arabidopsis IPI1/EMB175 included the *rps3-rps16* intergenic region that was conserved in maize (Hammani et al. 2016). Supporting the value of computationally predictions in revealing the action of a PPR protein, maize PPR103 bound to and stabilized the 5’ end of the processed *rps16* mRNA. A refined algorithm suggested that the mitochondrial *NAD2* transcript was the most likely target for AtIPI1 (Kobayashi et al. 2019); however, this finding is not likely relevant to EMB175/IPI1/PPR103 function since the protein is found in chloroplasts and not in mitochondria, at least under our experimental conditions (Fig. 4). We therefore focused on identifying potential chloroplast targets for NbIPI1 (Fig. 6a) using the FIMO tool, as previously described (Takenaka et al. 2013; Grant et al. 2011). Several potential target sites were identified including in the coding strands of *rpoC2* and *ndhB* transcripts (Fig. 6b) that contain sites edited in *N. benthamiana* (Fig. 1).

### NbIPI1 functions in chloroplast C-to-U RNA editing in N. benthamiana

**Fig. 7:**
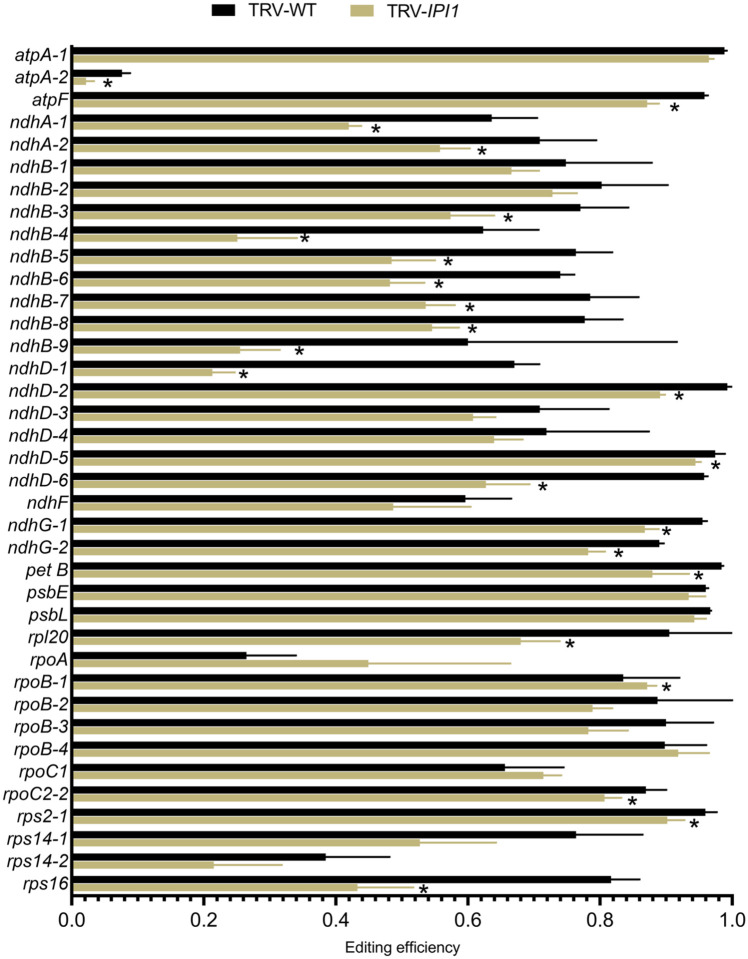
C-to-U editing in *IPI1*-silenced plants. RNA-seq analysis of *N. benthamiana* chloroplast transcripts confirms editing of all sites identified by Sanger sequencing and reveals reduced editing of several sites in *NbIPI1*-silenced chloroplasts. Editing efficiency was calculated by number of reads carrying the edited base as a proportion of the total number of reads for a given site. Error bars represent standard deviation of reads from individual libraries, with four replicates used for *NbIPI1*-silenced chloroplasts and three for the TRV-infected non-silenced controls. Asterisks denote *P*-value <0.05 as determined using a one-tailed *t*-test assuming unequal variance

To obtain a global picture of the RNA processing landscape in *N. benthamiana* chloroplasts, RNAseq analysis was conducted on total RNA from chloroplasts from leaves of *IPI1*-silenced or non-silenced control *N. benthamiana* plants. To ensure that enough chloroplasts were available for RNA isolation and subsequent cDNA library preparation, all chlorotic leaves from one silenced plant were pooled to produce one biological replicate. Samples from TRV-infected non-silenced control plants were similarly generated. The chloroplasts from control plants were phenotypically normal as observed by bright-field microscopy, whereas almost all chloroplasts isolated from *IPI1-*silenced mutant tissue were distinctively defective, with no thylakoid structures or starch granules apparent (Supplementary Figure S3), as expected from TEM observations (Fig. 4). Three control RNA-seq libraries (TRV-infected, non-silenced) and four test libraries (VIGS-*IPI1*) were used for Illumina MiSeq analysis (see Materials and Methods and Supplementary Figure S3). The reads had high quality mapping scores before and after the adapters were trimmed, ensuring that good quality contigs were subsequently mapped to the *N. benthamiana* chloroplast reference genome. The 250-nucleotide long sequenced reads were mapped to the *N. benthamiana* chloroplast reference genome curated by the Queensland University of Technology (https://sefapps02.qut.edu.au/benWeb/subpages/chloroplast.php) with no mismatch penalty to allow the detection of multiple editing events within the same transcript. Editing events were detected using an embedded SNP detection algorithm in the DNA Array Star workflow and are represented as the fraction of reads with an edited base out of the total reads (edited + unedited) for a given site. Additionally, reads that mapped to multiple locations were not excluded from subsequent analysis to allow the detection of potential SNPs on the inverted repeat strand of *ndhB*, a transcript that is heavily edited (Fig. 1). The editing efficiency at 23 of the 38 edited sites detected in this analysis transcripts was statistically significantly reduced in *IPI1-*silenced leaves compared to control leaves (Fig. 7). In contrast to the changes observed when *ISE2* was silenced, only the *rpoA* transcript was more edited in *IPI1*-silenced chloroplasts compared to the non-silenced controls. Thus, NbIPI1 may have roles in editing multiple chloroplast transcripts.

### Determining specificity between editing factors

**Fig. 8:**
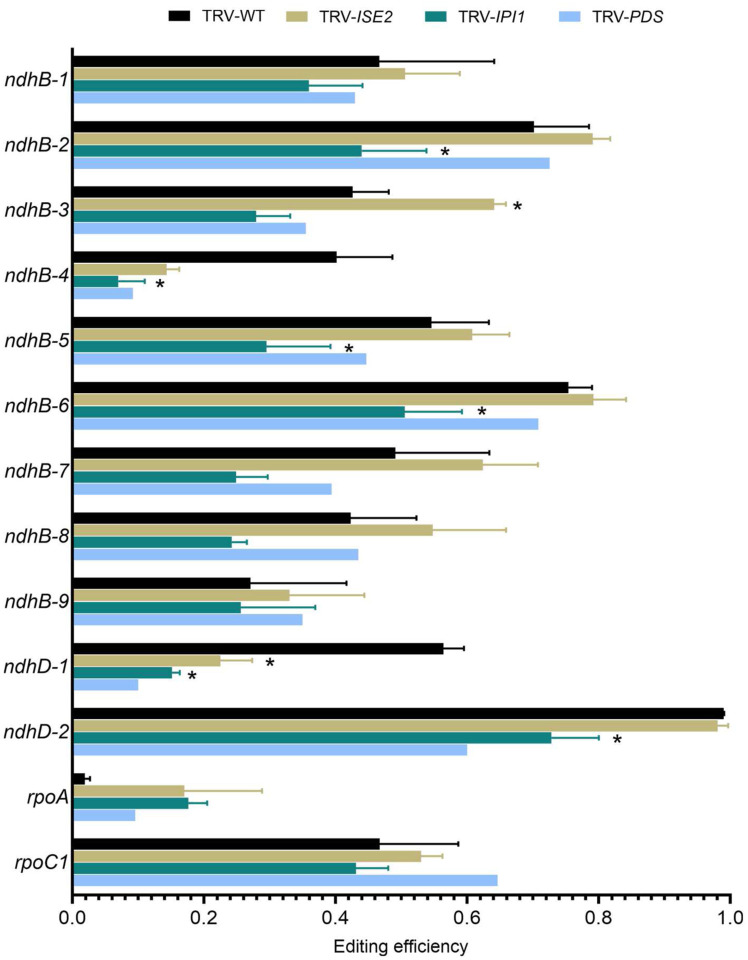
Possible specific editing activity of NbIPI1. RNA editing compared between *N. benthamiana* plants displaying chlorosis resulting from silencing *IPI1*, *ISE2* or *PDS* and TRV-infected, non-silenced controls by Sanger sequencing of transcripts. Results represent two non-silencing control biological replicates, three biological replicates for silencing *IPI1* or *ISE2*, and one biological replicate for silencing *PDS*. Error bars represent standard deviation for each replicate. Asterisks denote *P*-value <.05 as determined using a one-tailed Student’s *t*-test assuming unequal variance, pairwise comparisons were made between controls and silenced plants. Note that data for controls and TRV-*ISE2* are also presented in Figure 3

Since AtIPI1/EMB175 interacts with AtISE2 which was previously shown to be required for chloroplast C-to-U editing, it is possible that some defects in editing *IPI1*-silenced chloroplasts were due to indirect effects on ISE2 activity. In addition, general stress in chlorotic leaves is also known to have deleterious effects on C-to-U editing (Kakizaki et al. 2009; Tseng et al. 2013; Zhu et al. 2014). For these reasons and to confirm the results from the deep sequencing approach, we bulk sequenced select chloroplast transcripts from *IPI1*-, *ISE2*- or *PHYTOENE DESATURASE* (*PDS*)-silenced plants and compared the results to editing in non-silenced TRV-infected control plants. Sanger sequencing confirmed defects in editing in transcripts from *IPI1*-silenced leaves (Fig. 8). As expected, knockdown of *ISE2* or *PDS* in silenced plants also resulted in defective C-to-U editing and the defective editing of the *rpoA, ndhB-4*, and *ndhD-1* sites is likely a secondary effect of chloroplast dysfunction since these sites also had editing defects in other chlorotic leaves. Closer examination of editing defects at other sites revealed that the editing “signatures” caused by knockdown of *IPI1* or *ISE2* are distinct (Fig. 8). For example, the *ndhB-3* site is uniquely affected in *ISE2*-silenced plants and showed a surprising drastic increase in editing when *ISE2* expression was knocked down, while the *ndhB2–8* sites are uniquely affected in *IPI1*-silenced plants. Indeed, sites within the *ndhB* transcript *ndhB-2,−3,−5,−6,−7* and −*8* were specifically affected by knockdown of *NbIPI1*, consistent with our computational prediction (Fig. 6). This result indicates that while some editing defects may be due to a general stress response, each factor may differentially affect the editing reaction at specific sites within certain transcripts, and that *NbIPI1* is involved in C-to-U editing of chloroplast transcripts.

### NbIPI1 is necessary for the accumulation of chloroplast rRNA species

**Fig. 9:**
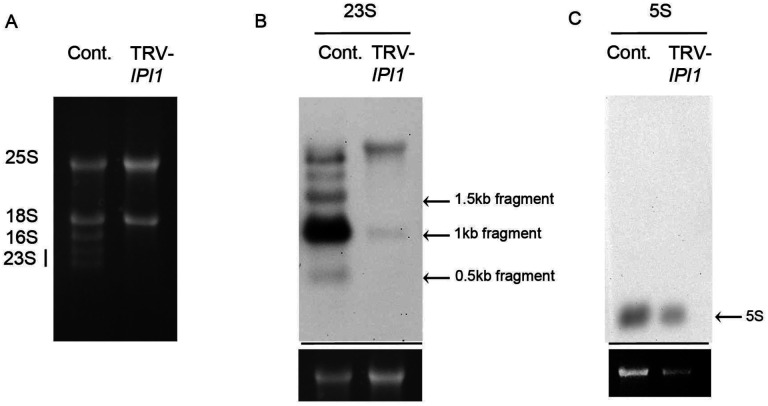
The effects of knockdown of *NbIPI1* expression on rRNA transcripts. (**A**) Total RNA from TRV-infected, non-silenced plants and *NbIPI1*-silenced plants stained with ethidium bromide. (**B**) Northern blot for 23S rRNA. Loading control (bottom panel) shown is from (**A**). (**C**) Northern blot for 5S rRNA and loading control (bottom panel). All contents of blots are shown, except for the loading controls

Knockdown of *NbIPI1* expression by TRV-mediated VIGS in led to severe leaf chlorosis and reduced chlorophyll content together with photosystem II quantum efficiency [(Ganusova et al. 2020) and Fig. 4)]. Similarly, maize *ppr103* mutant seedlings had albino leaves and did not continue development past the seedling stage (Hammani et al. 2016). RNA blot analysis revealed drastically reduced chloroplast ribosomal RNA (rRNA) levels in the *ppr103* albino leaves (Hammani et al. 2016). To test whether NbIPI1 may have a similar role in chloroplast rRNA biogenesis, we performed Northern blotting analysis for chloroplast rRNAs in *NbIPI1*-silenced *N. benthamiana* leaves using sequence-specific probes (Table S3). Defects in rRNA levels were severe enough to be observed on an agarose gel stained with ethidium bromide (Fig 9a). The Northern blots revealed that silencing *NbIPI1* led to major defects in chloroplast rRNA, with drastic reductions in the 23S, and minor decrease in 5S rRNAs compared to the rRNA levels in the TRV-containing non-silenced controls (Fig. 9b and c). These results suggest that IPI1’s role in rRNA processing is conserved in maize and *N. benthamiana,* although ribosome-associated transcripts were not among the top hits for NbIPI1 targets in our computational analysis (Fig. 6).

## DISCUSSION

*N. benthamiana* has emerged as model for elucidating plant responses to microbes, for various aspects of plant cell and molecular biology, and for protein expression in a variety of biotechnological settings (Goodin et al. 2008; Bally et al. 2018). This Australian native from the *Suaveolentes* section of *Nicotiana* is allotetraploid and it may have arisen from a hybridization event occurring about 5 MyA (Schiavinato et al. 2020). The *N. benthamiana* genome has been sequenced (Bombarely et al. 2012) and there are comprehensive collections of transcriptomic data (Nakasugi et al. 2013; 2014; Bally et al. 2015). Given its widespread use and evolutionary relationships to other members of the Solanaceous family, understanding the growth and development of *N. benthamiana* is important. Chloroplast development and function are essential for plant survival and, perhaps not surprisingly, mutants with defects in chloroplast often fail to complete embryogenesis or live past the seedling stage (Asakura et al. 2004; Bryant et al. 2011). Correct RNA processing for chloroplast gene expression is a critical aspect of chloroplast development, and much has been learnt about RNA metabolism in chloroplasts (Maier et al. 2008; Stern et al. 2010; Wang et al. 2021). We therefore set out to better characterize RNA processing in *N. benthamiana,* focusing on C-to-U RNA editing in chloroplast transcripts and the protein factors involved. We find that editing of *N. benthamiana* chloroplast transcripts is similar to that reported for our *Nicotiana* species, and the editing efficiency was dependent on leaf developmental stage (Figs. 1 and 2).

The results presented herein support a role for IPI1/PPR103 in rRNA processing, previously reported for its maize orthologue PPR103 (Hammani et al. 2016). As reported for PPR103, we find that NbIPI1 is involved in the processing of rRNA transcripts (Fig. 9). The results also extend NbIPI1’s role to C-to-U editing of chloroplast transcripts (Fig. 7 and 8). Our analysis of RNA editing in Nb*PDS*-silenced leaves suggests that NbIPI1 has a specific role in editing three sites within the *ndhB* transcript (Fig. 8). Most DYW PPR proteins have a single editing target and little redundancy between editing factors has been observed, probably due to the specificity with which each protein binds it target RNA (Barkan and Small 2014; Gutmann et al. 2020). Indeed, at least six PPR proteins have been identified as required for the Arabidopsis *ndhB* transcript, with a single site being identified as the target for each protein (Hammani et al. 2009; Okuda et al. 2009; Okuda et al. 2010; Hayes et al. 2013; Fu et al. 2022;). This contrasts with Arabidopsis QED1 that edits 1 site in each of five Arabidopsis chloroplast transcripts, an usually large number of sites (Wagoner et al. 2015). NbIPI1 potentially specifically targets 3 sites in the *ndhB* transcript from *N. benthamiana* chloroplasts, and further analysis would determine in sites in other edited transcripts (Fig. 7) are specific NbIPI1 targets. Indeed, the computational prediction of targets suggests that editing of other could potentially involve NbIPI1 (Fig. 6).

### The role of the DYW motif in editing

Nucleus-encoded RNA processing factors that are organelle-targeted are responsible for RNA editing and consistent with this, defects in chloroplast translation do not affect RNA editing (Zeltz et al. 1993). Thus far, nuclear-encoded PPR proteins have been classified as site-specific trans-factors involved in RNA editing (Mach 2009). The DYW domain’s predicted structure resembles that of cytidine deaminases which bind zinc as part of their catalytic activity (Salone et al. 2007; Iyer et al. 2011). Mutations within the DYW domain of DYW1, a PPR protein that is similar to IPI1, greatly impair both zinc-binding and RNA editing, indicating that the DYW domain may confer cytidine deaminase activity (Okuda et al. 2009). Interestingly, the specific C-terminal amino acid residues DYW or any variation thereof is not found at the C-terminus of the maize IPI1 orthologue, PPR103 (supplemental Fig. S2), and no editing events that could be attributed to PPR103 were disrupted in the *ppr103* mutant (Hammani et al. 2016). This observation suggests that the specific DYW triad or its variants contribute to the editing reaction at several chloroplast editing sites. Several recent studies have clarified the importance of the DYW domain in RNA editing. Expression of a moss (*Physcomitrium patens*) PPR protein, PPR65, in *E. coli* was sufficient to edit its corresponding mitochondrial site, ccmFCeU103PS, with 70–100% efficiency (Oldenkott et al. 2019). A second moss PPR protein, PPR56, was also able to edit both its targets, nad4eU272SL and nad3eU230SL in the *E. coli* system with the same efficiencies observed *in planta*. *In vitro* assays demonstrated that purified recombinant moss PPR65 could successfully perform C-to-U editing of its synthetically generated target RNA, and that the editing activity required zinc and was enhanced by ATP or nonhydrolyzable nucleotide analogs, giving a glimpse into the editing mechanism of DYW proteins (Hayes and Santibanez 2020). Together these studies advanced the idea that DYW proteins could independently carry out C-to-U editing. The mechanism of DYW domain has been more fully revealed by the structural details gleaned from the recent crystal structure of the DYW domain of *Arabidopsis thaliana* OTP86 protein that specifically edits a site in the *rps14* transcript (Takenaka et al. 2021). Those studies revealed a cytidine deaminase fold and a DYW domain containing zinc atoms that were critical to editing activity through the regulation of a “gated zinc shutter”. *In vitro* RNA editing assays confirmed the importance of highly conserved residues to catalysis and highlighted the importance of the coordinating Zn ions for catalysis (Zn1) or stability of the DYW motif (Zn2) (Takenaka et al. 2021), supporting findings from previous mutational studies with other DYW proteins (Hayes et al. 2013; Boussardon et al. 2014; Hayes et al. 2015; Oldenkott et al. 2019; Hayes and Santibanez 2020). Our data support the importance of the DYW triad for editing as they demonstrate that the presence of a variant of the DYW domain in NbIPI1 was sufficient to confer a role in editing (Figs. 7 and 8).

### NbISE2 and NbIP1 functions in RNA editing in N. benthamiana

ISE2 is an evolutionarily conserved chloroplast-localized RNA helicase that has many roles in RNA processing (Carlotto et al. 2016; Bobik et al. 2017). ISE2 is required for C-to-U RNA editing at multiple sites in Arabidopsis (Bobik et al. 2017), and the maize ortholog was identified in multi-protein complexes that edited the C473 site in maize *ndhA* transcripts (Sandoval et al. 2019). The ISE2-containing editing complex from maize also contained the non-PPR editing factors RIP1/MORF8 and RIP9 as well as ORRM, containing an RNA-Recognition Motif (RRM) and OZ1, a RanBP2-type Zn finger protein. Several PPR proteins including six P-type PPR proteins previously not known to be involved with RNA editing were also identified (Sandoval et al. 2019). The interaction of ISE2 with other editing factors and PPR proteins is consistent with the identification of several RNA binding proteins including IPI1 as interacting with ISE2 in a yeast two-hybrid screen (Bobik et al. 2019). While Arabidopsis reduced ISE2 levels led to significantly reduced editing specifically at three sites (*rpoB-338*, *rpoB-551* and *rps14–149*) (Bobik et al. 2017), silencing *ISE2* in *N. benthamiana* significantly reduced editing at only the *ndhD-1* site (Fig. 3) and unexpectedly increased editing *rpoB-1* (*rpoB-338* in Arabidopsis) and *rpoB-2* (*rpoB-473* in Arabidopsis) sites (Fig. 3 and Table S1). Thus, ISE2 may have a conserved role in editing the rpoB-1 (nt 338). Interestingly, rpoB was one of the top predicted targets for NbIPI1 binding (Fig. 6) and while editing of numerous sites was decreased when *NbIPI1* was silenced by VIGS, editing of *rpoB-1* was uniquely significantly increased (Figure 7). This suggests that IPI1 and ISE2 may function as part of an editing complex for at least this site. Other findings suggest, however, that there are unique signatures of editing defects observed in *IPI1* compared to *ISE2-*silenced leaves that are likely not the result general stress. For example, *ndhB-2* and *ndhB-8* are uniquely affected in *IPI1-*silenced leaves and *ndhB-3* is uniquely affected in *ISE2-*silenced leaves (Fig. 8). Most of the sites that are affected by loss of IPI1 exhibit a reduction in editing efficiency, while these same sites exhibit a slight (but not statistically significant) increase in editing efficiency in *ISE2-*silenced leaves. In fact, a general trend for editing defects is an increase observed in *ISE2* silenced-leaves and a decrease observed in *IPI1-*silenced-leaves (Fig 5). These results may suggest that ISE2 and IPI1 perform antagonistic roles in editing some *N. benthamiana* chloroplast transcripts.

## Data Availability

The dataset supporting the conclusions of this article is available in the NCBI Sequence Read Archive (SRA) repository, PRJNA826083 and https://www.ncbi.nlm.nih.gov/bioproject/PRJNA826083.

## KEY MESSAGE:

The RNA helicase NbISE2 and PPR protein NbIPI1 are involved in chloroplast C-to-U RNA editing in *Nicotiana benthamiana*, and they may act together at some sites but antagonistically at others.
